# Low IL‐23 levels in peripheral blood and bone marrow at diagnosis of acute leukemia in children increased with the elimination of leukemic burden

**DOI:** 10.1111/jcmm.16772

**Published:** 2021-07-08

**Authors:** Archontis Zampogiannis, Christina Piperi, Margarita Baka, Iliana Zoi, Athanasios G. Papavassiliou, Maria Moschovi

**Affiliations:** ^1^ Pediatric Hematology‐Oncology Unit Medical School "Agia Sophia" Children's Hospital National and Kapodistrian University of Athens Athens Greece; ^2^ Department of Biological Chemistry Medical School National and Kapodistrian University of Athens Athens Greece; ^3^ Department of Pediatric Hematology‐Oncology "P&A Kyriakou" Children's Hospital Athens Greece

**Keywords:** acute leukemia, acute lymphoblastic leukemia, acute myeloid leukemia, children, IL‐23

## Abstract

IL‐23 is an IL‐12 cytokine family member with pleiotropic functions that regulates tumour growth in various cancer types, exhibiting both anti‐tumorigenic and pro‐tumorigenic properties. Preclinical studies have shown a potential anti‐leukemic action on childhood B‐ALL cells. The study involved 65 children with acute leukemia [59 patients with acute lymphoblastic leukemia (ALL) and 6 patients with acute myeloid leukemia (AML)] and 27 healthy controls. Using an enzyme‐linked immunosorbent assay, we aimed to determine the IL‐23 levels in the peripheral blood (PB) and bone marrow (BM) of patients at diagnosis and at the end of the induction therapy (EIT). PB IL‐23 levels were lower in leukemia patients compared to the healthy controls. In all acute leukemia patients, IL‐23 levels were significantly lower at diagnosis both in PB (*P* = .015) and in BM (*P* = .037) compared to the PB and BM concentrations at the EIT. The same pattern was present in both subgroups of ALL and AML patients. The high leukemic burden at diagnosis was related with lower IL‐23 levels, which were increased with the disease remission. Considering the anti‐leukemic potential of this cytokine, the elevation of the IL‐23 concentration at the disease remission indicates a beneficial role of IL‐23 in paediatric acute leukemia.

## 1 INTRODUCTION

Acute leukemia represents the most common malignancy in childhood, accounting for approximately one‐fourth of all malignancies.^1^ Acute lymphoblastic leukemia (ALL) is the most common (about 80%) type of leukemia in this age group, deriving from the aberrant clonal proliferation of B‐lineage or T‐lineage lymphoblasts.^2^ In the last decades, major advances have been made in the treatment of childhood ALL, improving the cure rates from 10% to approximately 90%.^3^ However, approximately 15%‐20% of children experience disease recurrence.^4^

The role of inflammation and aberrant cytokine signalling on oncogenesis has gained interest in both solid tumours and haematological malignancies.^5^, ^6^ Blood cells and their bone marrow‐based progenitors are very responsive to their environment, producing several immunomodulators. Among them, cytokines are of primary importance, regulating the functions of B and T lymphocytes as well as natural killer cells, and inducing lymphopoiesis.^7^, ^8^, ^9^ It has been proposed that aberrant cytokine production supplements genetic abnormalities during leukemogenesis, providing leukemic cells with a survival and proliferation advantage, attributed to dysregulated cell cycle and impairment of the normal immune surveillance.^10^ Both cytokine over‐expression by leukemic and immune cells, as well as the inadequate cytokine expression, can promote leukemogenesis.^10^, ^11^, ^12^ The aberrant cytokine secretion in leukemia patients is normalized in complete remission, suggesting an autonomous pattern of cytokine expression that is dependent on leukemic activity.^13^

Moreover, the balance between pro‐inflammatory and anti‐inflammatory cytokines plays an essential role on how the inflammatory cells are involved in tumour growth.^14^, ^15^ Malignant cells are able to evade the immune system surveillance by using a variety of methods, including production and secretion of soluble factors that suppress specific anti‐tumour immune responses. There is evidence that the inhibition of anti‐tumour immune responses, and thus, the promotion of tumour growth, is related to the shift from Th1 lymphocyte responses to Th2 responses in the tumour microenvironment.^16^ Th1 cells produce cytokines, such as IL‐12, IFN‐γ and TNF‐α, that are pro‐inflammatory and promote cell‐mediated immunity to act versus tumour cells, while the Th2 cytokines exert anti‐inflammatory and immunosuppressive properties. The Th1 and Th2 cytokines act antagonistically and the shift in favour of Th1 facilitates the anti‐tumour immune responses.^14^ In solid tumours, as well as in acute and chronic leukemias microenvironment, there is a shift towards the Th2 cytokine secretion by immune and tumour cells.^17^, ^18^ Indeed, in several cancer types, the IL‐12 levels, that reflect the Th1 immune response, were significantly lower in patients with advanced disease in comparison with patients with moderate disease.^14^, ^19^ The IL‐12 family members are mainly involved in the promotion and maintenance of Th1 cell differentiation and high IL‐12 levels have been associated with less advanced malignancies and better prognosis.^19^ Moreover, in some cancer types, the response to therapy was followed by an elevation of the initially low serum IL‐12 levels.^14^, ^20^

IL‐23, an IL‐12 cytokine family member, exhibits structural similarities with IL‐12 but substantially distinct biological role.^21^, ^22^ IL‐23 is a heterodimeric cytokine that is comprised of the IL‐12p40 subunit, common with IL‐12, and the IL‐23‐specific p19 subunit. The IL‐23 receptor is composed of the IL‐12Rβ1 chain, that is shared with the IL‐12 receptor, and the exclusive IL‐23R chain.^21^, ^23^ IL‐23 is produced by myeloid dendritic cells and by type 1 macrophages in response to microbial and host immune stimuli, such as Toll‐like receptors and IFNs. IL‐23 activates the same Jak/STAT signalling molecules as IL‐12, namely Jak2, Tyk2 kinases and STAT1, −3, −4 and −5 transcription factors. In contrast to IL‐12 that predominantly induces the activation of STAT4, the main transcription factor induced by IL‐23 is STAT3.^24^

IL‐23 stimulates the proliferation of Th1 memory cells and promotes the proliferation of Th17 cells, a subset of T cells that expresses IL‐17, which has a crucial role in the development of autoimmune inflammation.^23^, ^25^ Also, IL‐23 modulates IgM and IgG secretion in plasma cells by upregulating the IgM secretion, while inhibiting the IgG secretion, suggesting that IL‐23 is mainly involved in primary immune responses.^26^ The potent anti‐tumour activity of the IL‐12 family cytokines has been put under investigation, and while the anti‐tumour activity of IL‐12 and IL‐27 has been clearly established, the role of IL‐23 appears to be controversial.^22^ In this context, we hypothesized that IL‐23 expression profile may be different in states of leukemic burden, and contribute to disease pathogenesis.

Therefore, the aim of this study was to determine the levels of IL‐23 in the peripheral blood (PB) and the BM of children with acute leukemia, reveal any potential correlations with clinicopathological features and detect any significant differences between diagnosis and the end of the induction therapy (EIT).

## 2 MATERIALS AND METHODS

### 2.1 Patients

Our study involved 65 children with acute leukemia, diagnosed and treated in the University Pediatric Hematology‐Oncology Unit of the “Agia Sophia” Children's Hospital and the Pediatric Hematology‐Oncology Unit of the “P&A Kyriakou” Children's Hospital in Athens, Greece, from January 2011 to May 2016, and 27 children admitted to the hospital for minor surgical procedures, serving as controls.

The patient cohort was comprised of 65 children (34 males and 31 females) aged from 11 months to 18 years old, with median age 4.9 years old (2.9‐9.8) (Table 1). Fifty‐nine (59) children were diagnosed with ALL and 6 children were diagnosed with AML. Among patients with ALL, 50 children were diagnosed with B‐cell ALL, 6 children were diagnosed with T‐cell ALL, 2 children were diagnosed with Burkitt‐cell acute leukemia and one child with a blast crisis relapse of Philadelphia chromosome‐positive CML. Children with ALL, including the one with the blast crisis relapse of CML, were treated according to the ALLIC BFM 2009 protocol. The two cases of Burkitt‐cell acute leukemia were treated according to the FAB‐LMB 96 protocol. Patients with AML received therapy according to the AML BFM 2004 and AML BFM 2012 protocol.

**TABLE 1:** Demographic and clinicopathological characteristics of patients and controls

Patients	ALL	B‐ALL	T‐ALL	AML	Controls
Total number	59	53	6	6	27
Gender (male/female) (n)	34/31	25/28	6/0	3/3	14/13
Age					
<6 y (n)	35	32	3	1	15
>6 y (n)	24	21	3	5	12
WBC count					
<20.000/μL (n)	37	37	0	3	
>20.000/μL (n)	22	16	6	3	
LDH					
<1000 IU/L (n)	25	25	0	4	
>1000 IU/L (n)	30	24	6	2	
MRD Result (EIT)					
Negative (n)	47	44	3	1	
Positive (n)	12	9	3	4	

Abbreviations: EIT, end of the induction therapy; LDH, lactate dehydrogenase; MRD, minimal residual disease at the end of the induction therapy; n, number of patients/controls; WBC, white blood cells.

The control group consisted of 27 children (14 males and 13 females), aged from 2 to 13 years old, with median age 5.5 years old (3.5‐10), with detailed medical history, and clinical examination confirming absence of inflammatory or any infectious diseases for a month prior the sample collection, and without receiving any medication.

The study was conducted according to the guidelines of the Declaration of Helsinki and approved by Ethics Committee of the “Agia Sofia” and “P&A Kyriakou” Children's Hospital, Athens (Protocol number: 16483/25‐07‐13). An informed consent was obtained from all patients involved in the study.

### 2.2 PB and BM sample collection and IL‐23 determination

Peripheral blood samples and BM aspirates were collected from children with acute leukemia before the initiation of treatment (day 0) and at the EIT (Day 33 for patients with ALL and Day 28 for patients with AML, respectively). In cases of Burkitt‐cell acute leukemia, samples were collected at diagnosis and after the completion of COPADM1 scheme of induction therapy.

Bone marrow was collected into EDTA vacutainer tubes and PB in serum‐separating tubes. PB serum and BM plasma were obtained by centrifugation, divided into aliquots, and stored at −80℃ until assayed. Repeated freeze‐thaw cycles were avoided for all samples.

IL‐23 levels were determined in 60 PB and 59 BM pretreatment samples, 28 PB and 35 BM samples after the completion of the induction therapy and 27 healthy controls. The alterations in IL‐23 levels between diagnosis and the EIT were estimated in PB and BM in children for which both samples (at diagnosis and at the EIT) were available. IL‐23 concentration in PB serum and in BM plasma was measured by enzyme‐linked immunosorbent assay (ELISA) for quantitative detection kits (Human IL‐23 Platinum ELISA, eBioscience) according to the manufacturer's instructions.

The analytical sensitivity was 5.0 pg/mL and the assay range is 31.3‐2000 pg/m. The inter‐assay coefficient of variation was 4.5% and intra‐assay, 6.7%. The absorbance value at 450‐nm wavelength was read with a microplate reader (VersaMax Tunable, Molecular Devices). Each measurement represents the average of triplicate wells.

### 2.3 Statistical analysis

Continuous variables normally distributed are presented as means (standard deviation (SD)), whereas non‐normally distributed variables are presented as median (25th, 75th percentile). The Kolmogorov‐Smirnov goodness‐of‐fit test was used to assess the distribution of each variable. Comparisons between continuous variables were performed with Student's *t* test and paired *t* test or Mann‐Whitney and Wilcoxon test depending on normality of distribution.

Statistical analyses were performed with the STATA software (version 13.0, College Station™). All *P*‐values were two‐sided and a cut‐off value of *P* < .05 was set to denote statistical significance.

## 3 RESULTS

### 3.1 IL‐23 levels were detected in PB and BM samples of ALL and AML patients

IL‐23 pretreatment levels (in pg/mL) were detected in PB and BM of ALL [PB: 11.32 (6.21‐19.38), BM: 12.08 (4.39‐27.70)] and AML patients [PB: 14.72 (11.23), BM: 6.61 (5.56‐7.64)]. The pretreatment IL‐23 levels did not differ significantly between ALL patients and AML patients neither in PB (*P* = .834) nor in BM (*P* = .531). In ALL patients, no statistically significant differences were observed between the pretreatment IL‐23 levels in B‐ALL and T‐ALL patients neither in PB [11.81 (6.14‐21.49) vs 10.31 (7.25), *P* = .554] nor in BM [12.72 (4.17‐28.20) vs 10.04 (6.46‐12.08), *P* = .933].

Evaluation of demographic and clinical data of ALL and AML patients in correlation with pretreatment PB and BM IL‐23 levels revealed no statistical significance between subgroups for age, gender, as well as for white blood cell count (WBC), lactate dehydrogenase (LDH) and the minimal residual disease (MRD) result at the EIT, as shown in Table 2.

**TABLE 2:** Evaluation of IL‐23 concentration (in pg/mL) in peripheral blood (PB) and bone marrow (BM) in correlation with the demographic and clinical data of patients

Disease		ALL				AML			
		PB IL‐23	*P*‐value	BM IL‐23	*P*‐value	PB IL‐23	*P*‐value	BM IL‐23	*P*‐value
Gender	Males	11.81 (6.21‐17.84) (n = 30)	.715	12.04 (4.50‐25.70) (n = 27)	.915	19.08 (15.35) (n = 3)	.401	21.68 (28.15) (n = 3)	.402
	Females	10.77 (6.67‐23.22) (n = 24)		13.01 (4.17‐29.20) (n = 26)		10.36 (4.75) (n = 3)		6.43 (1.08) (n = 3)	
Age	<6 y	10.22 (6.21‐15.58) (n = 31)	.309	8.67 (4.17‐24.59) (n = 32)	.287	15.15 (n = 1)	‐	7.12 (n = 1)	‐
	>6 y	16.45 (6.07‐30.0) (n = 23)		16.84 (6.33‐27.70) (n = 21)		14.63 (12.55) (n = 5)		6.10 (5.56‐7.64) (n = 5)	
WBC	<20 000/μL	11.32 (6.07‐17.47) (n = 33)	.818	12.72 (4.17‐29.2) (n = 33)	.826	10.36 (4.75) (n = 3)	.401	6.43 (1.08) (n = 3)	.402
	>20 000/μL	10.76 (7.13‐18.61) (n = 20)		10.51 (5.43‐21.92) (n = 20)		19.08 (15.35) (n = 3)		21.68 (28.15) (n = 3)	
LDH	<1000 IU/L	8.54 (4.39‐13.43) (n = 20)	.271	13.0 (3.77‐28.7) (n = 24)	.947	16.76 (13.40) (n = 4)	.586	6.87 (5.83‐30.89) (n = 4)	.355
	>1000 IU/L	12.67 (7.49‐19.38) (n = 30)		12.04 (4.17‐27.70) (n = 27)		10.61 (6.42) (n = 2)		5.46 (2.35) (n = 2)	
MRD Result at the EIT	Negative	11.32 (4.95‐19.38) (n = 43)	.982	8.53 (4.17‐29.2) (n = 42)	.554	15.15 (n = 1)	‐	7.12 (n = 1)	‐
	Positive	8.85 (6.76‐19.97) (n = 11)		15.52 (9.17) (n = 11)		15.44 (14.34) (n = 4)		6.60 (4.68‐30.89) (n = 4)	

Note

IL‐23 concentration is presented as median value (25th, 75th percentile) in cases of non‐normal distribution and as mean value (standard deviation (SD)) in cases of normal distribution.

Abbreviations: ALL, acute lymphoblastic leukemia; AML, acute myeloid leukemia; BM, bone marrow; EIT, end of the induction therapy; LDH, lactate dehydrogenase; MRD, minimal residual disease at the end of the induction therapy; n, number of patients; PB, peripheral blood; WBC, white blood cells.

### 3.2 Lower IL‐23 levels in PB of ALL patients at diagnosis compared to healthy controls

**FIGURE 1:**
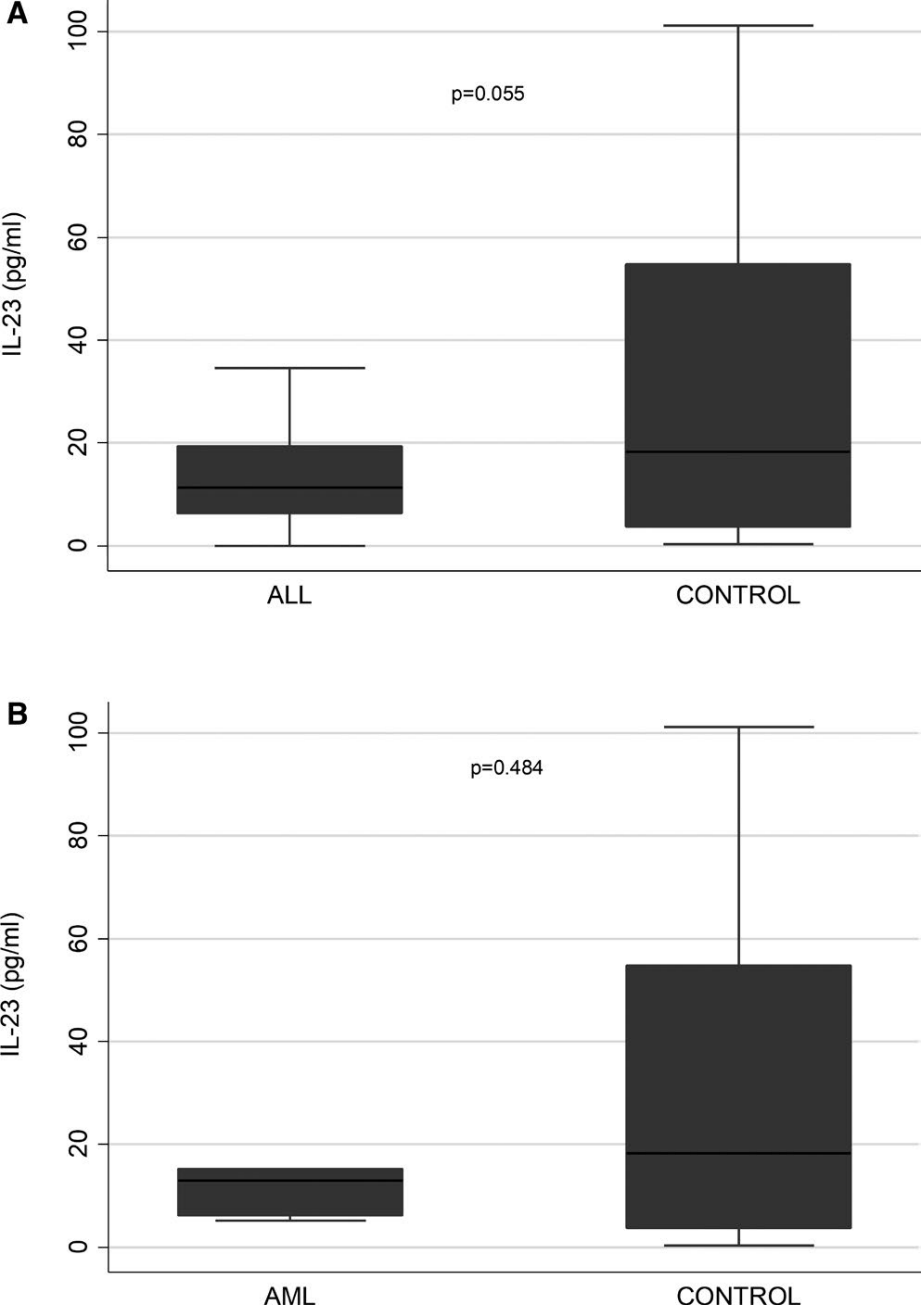
A, Lower IL‐23 levels in peripheral blood (PB) of acute lymphoblastic leukemia (ALL) patients (n = 54) at diagnosis compared to healthy controls (n = 27). B, Lower IL‐23 levels in PB of acute myeloid leukemia (AML) patients (n = 6) at diagnosis compared to healthy controls (n = 27). Box plots represent the first (25%) and the third (75%) quartile of the distribution. The cross‐line denotes the median, and the lower and upper whiskers represent the 1st and the 99th percentiles, respectively

In all leukemia patients, PB IL‐23 levels at diagnosis were lower compared to healthy controls [11.38 (6.14‐18.61) vs 18.22 (3.60‐54.79)] with a marginal statistical significance (***P* = .058**). In ALL patients, the median IL‐23 concentration was 11.32 (6.21‐19.38), while in healthy controls, it was 18.22 (3.60‐54.79), (***P* = .055**) (Figure 1A). In AML patients, no statistically significant differences were found between the IL‐23 levels at diagnosis and healthy controls [14.72 (11.23) vs 18.22 (3.60‐54.79), *P* = .484] (Figure 1B). No statistically significant differences were found between IL‐23 levels at the EIT of ALL patients and the healthy controls [34.1 (22.35) vs 18.22 (3.60‐54.79), *P* = .372].

### 3.3 Lower IL‐23 levels in PB and BM at diagnosis compared to the EIT in ALL and AML patients

**FIGURE 2:**
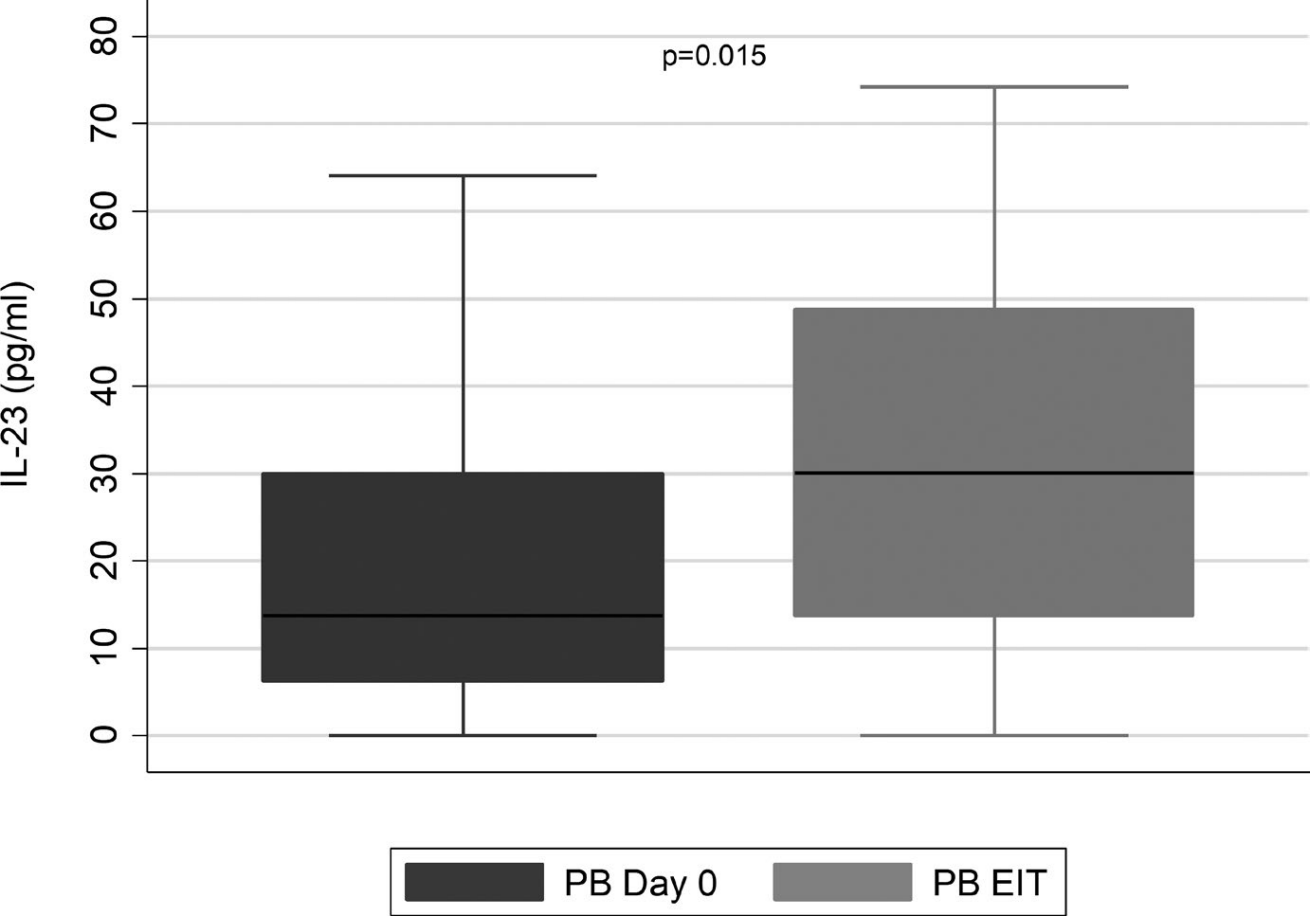
Lower IL‐23 levels in peripheral blood (PB) in acute lymphoblastic leukemia (ALL) patients (n = 26) at diagnosis (Day 0) compared to the end of the induction therapy (EIT). Box plots represent the first (25%) and the third (75%) quartiles of the distribution. The cross‐line denotes the median, and the lower and upper whiskers represent the 1st and the 99th percentiles, respectively

In all leukemia patients, PB IL‐23 levels at diagnosis were significantly lower than IL‐23 levels at the EIT [13.71 (6.21‐30) vs 32.79 (21.72), ***P* = .015**]. In ALL patients, PB IL‐23 levels at diagnosis were significantly lower than IL‐23 levels at the EIT [13.71 (6.21‐30) vs 32.79 (21.72), ***P* = .015**] (Figure 2). Regarding the different ALL subtypes, in B‐ALL patients, PB IL‐23 levels at diagnosis were significantly lower than at the EIT [10.41 (5.58‐30) vs 30.51 (20.62), ***P* = .039**]. The same pattern was also observed in T‐ALL patients [18.61 (1.09) vs 60.1 (19.94)] but without reaching statistical significance (*P* = .179) possibly because of the small number of T‐ALL patients.

**FIGURE 3:**
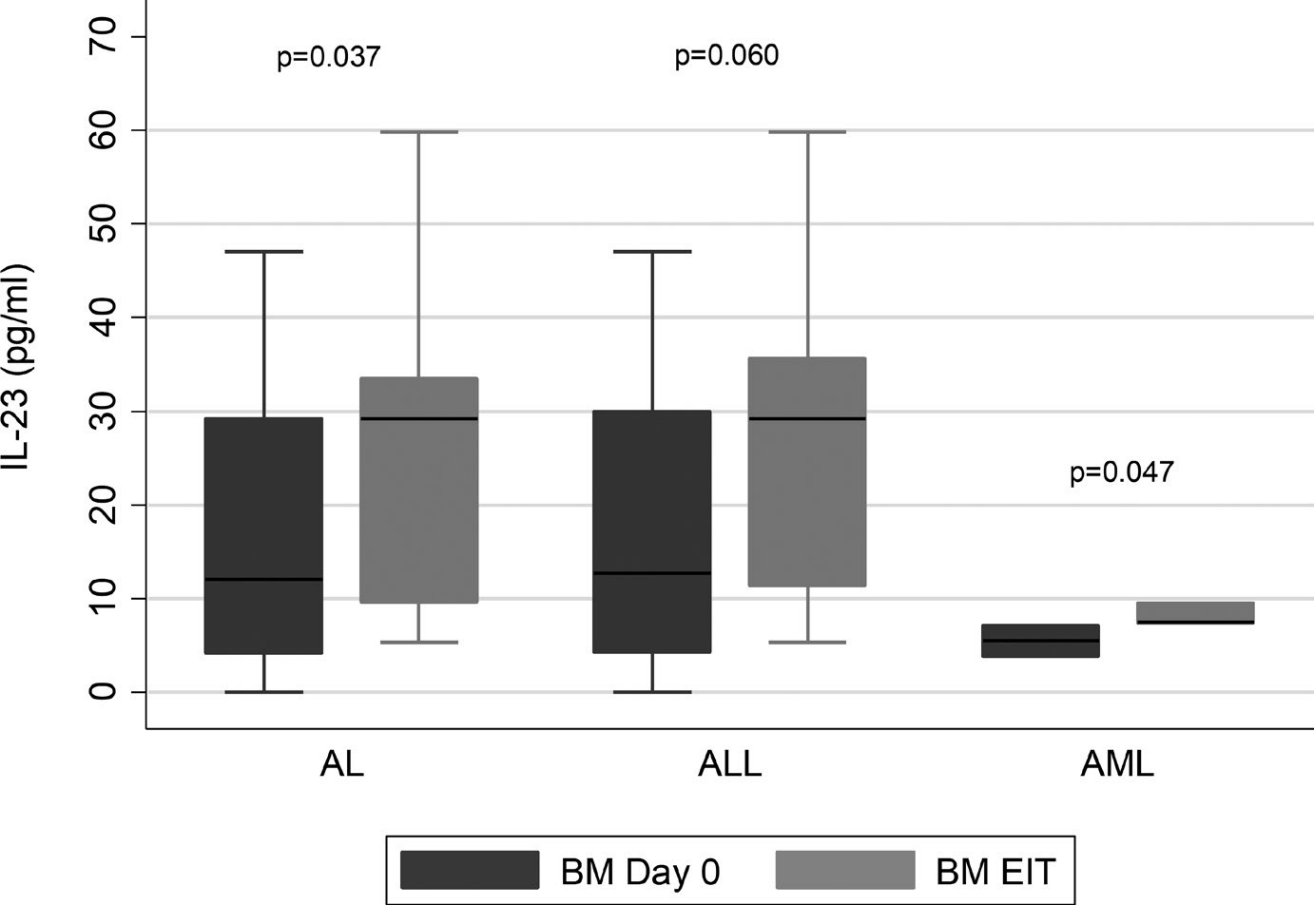
Lower IL‐23 levels in bone marrow (BM) in all leukemia (AL) patients (n = 35), acute lymphoblastic leukemia (ALL) patients (n = 32) and acute myeloid leukemia (AML) patients (n = 3) at diagnosis (Day 0) compared to the end of the induction therapy (EIT). Box plots represent the first (25%) and the third (75%) quartiles of the distribution. The cross‐line denotes the median, and the lower and upper whiskers represent the 1st and the 99th percentiles, respectively

In all leukemia patients, BM IL‐23 concentration at diagnosis was significantly lower than the BM concentration at the EIT [12.04 (21.53‐25.87) vs 29.2 (9.67‐33.48), ***P* = .037**] (Figure 3).

In ALL patients, lower BM IL‐23 concentration was observed at diagnosis compared to the EIT BM concentration, with a marginal significance observed [12.73 (4.33‐30.0) vs 29.2 (11.4‐35.6), (***P* = .060**)] (Figure 3). Regarding the ALL subtypes, lower BM IL‐23 levels at diagnosis were observed in both B‐ALL patients [14.48 (4.17‐30.81) vs 29.2 (11.55‐33.48), *P* = .147] and T‐ALL patients [8.85 (2.87) vs 33.41 (24.52), *P* = .189] compared to the EIT but without reaching a statistical significance.

In AML patients, the BM IL‐23 concentration at diagnosis was significantly lower than the EIT BM concentration [5.49 (1.66) vs 8.10 (1.25), ***P* = .047**] (Figure 3).

The comparison between the IL‐23 concentration at diagnosis and at the EIT in PB and BM is shown analytically in Table 3.

**TABLE 3:** Evaluation of IL‐23 concentration (in pg/mL) in peripheral blood (PB) and bone marrow (BM) at diagnosis and at the end of the induction therapy (EIT) in acute lymphoblastic leukemia (ALL) and acute myeloid leukemia (AML) patients

	PB IL‐23				BM IL‐23			
	n	Diagnosis	EIT	*P*‐value	n	Diagnosis	EIT	*P*‐value
All leukemias	26	13.71 (6.21‐30)	32.79 (21.72)	.**015**	35	12.04 (21.53‐25.87)	29.2 (9.67‐33.48)	.**037**
ALL	26	13.71 (6.21‐30)	32.79 (21.72)	.**015**	32	12.73 (4.33‐30.0)	29.2 (11.4‐35.6)	.060
B‐ALL	24	10.41 (5.58‐30)	30.51 (20.62)	.**039**	29	14.48 (4.17‐30.81)	29.2 (11.55‐33.48)	.147
T‐ALL	2	18.61 (1.09)	60.1 (19.94)	.179	3	8.85 (2.87)	33.41 (24.52)	.189
AML	0				3	5.49 (1.66)	8.10 (1.25)	.**047**

Note

IL‐23 concentration is presented as median value (25th, 75th percentile) in cases of non‐normal distribution and as mean value (SD) in cases of normal distribution. Bold values indicate significance.

Abbreviations: ALL, acute lymphoblastic leukemia; AML, acute myeloid leukemia; B‐ALL, B‐cell acute lymphoblastic leukemia; BM, bone marrow; EIT, end of the induction therapy; n, number of patients; PB, peripheral blood; T‐ALL, T‐cell acute lymphoblastic leukemia.

## 4 DISCUSSION

In order to determine the role of Th17 immunomodulatory cytokine, IL‐23 in children with acute leukemia, we estimated its concentration in PB and BM at diagnosis as well as at the EIT compared to healthy controls. Our data demonstrate significantly lower IL‐23 levels in PB and BM of acute leukemia patients at diagnosis compared to the respective values at the EIT. To our knowledge, this is the first study that detects alterations in IL‐23 levels after the application of chemotherapy in children with acute leukemia.

Of note, lower IL‐23 levels were observed in acute leukemia patient's PB at diagnosis compared to controls, without however reaching statistical significance. No differences were observed between controls and leukemia patients at the EIT, both in ALL and AML patients. In children with ALL, lower IL‐23 concentration was observed in both PB and BM at diagnosis compared to the EIT. However, a statistically significant difference was observed only in PB. It is worth to note that in BM of ALL patients, IL‐23 levels were also clearly lower at diagnosis compared to the EIT. Similar results were observed in AML patients, where IL‐23 concentration was significantly lower in the BM at diagnosis than at the EIT.

The role of IL‐23 in paediatric leukemia has been previously investigated by the Cocco et al, providing strong evidence of the anti‐leukemic potential of this cytokine. The ability of IL‐23 to inhibit B‐ALL growth was studied both in vitro by treating primary B‐ALL cells with IL‐23 and in vivo using SCID/NOD mice injected with 697 cell line expressing IL‐23R. It was shown that IL‐23 directly dampens tumour growth through the induction of apoptosis and inhibition of leukemic cell proliferation.^22^, ^24^ The mechanism by which IL‐23 exerts its anti‐leukemic properties is related to the regulation of microRNAs that regulate the expression of several genes and cellular functions, essential for tumorigenesis, including apoptosis, proliferation and differentiation. In childhood B‐ALL cells, IL‐23 induces the upregulation of miR15a, resulting in downregulation of BCL‐2 and Cyclin D1, which further affect apoptosis and proliferation.^22^ IL‐23 has been investigated in several tumours where it was shown to exert a tumour‐suppressing action upon overexpression in vitro and in vivo.^27^, ^28^, ^29^, ^30^ The anti‐tumour and anti‐metastatic effects of IL‐23 are similar to those of IL‐12 and seem to be mediated by CD8^+^ cells.^31^ The lower IL‐23 levels in leukemic patients compared to controls indicate that the IL‐23 downregulation is involved in leukemic cell expansion. Moreover, the elevation of IL‐23 concentration at the EIT indicates a beneficial effect of the cytokine considering its anti‐leukemic potential.

Previous studies on IL‐23 have demonstrated conflicting results in respect of its expression in cancer and its biological role, with only few investigating the IL‐23 expression in acute leukemias. El‐Maadawy et al^32^ have reported an elevation of IL‐23 concentration in paediatric ALL patients compared to controls. In adult B‐ALL and AML, increased levels of Th‐17 related cytokines, including IL‐23, have been detected in the PB and BM of patients.^33^, ^34^ Our study is the first that demonstrates low IL‐23 expression at diagnosis of paediatric acute leukemia.

Most cancer studies demonstrate that IL‐23 functions as a mediator of the Th17 immune response, exerting a critical role in Th17 cell expansion and the maintenance of the Th17 phenotype.^35^ While it is well‐documented that the Th1 immune response promotes cell‐mediated immunity and the Th2 response exerts an immunosuppressive effect, the role of Th17 cells in promoting or protecting from tumour progression has not been elucidated and is currently under debate.

The findings of our study are in accordance with the study of Karmali et al^36^ where CLL patients exhibit lower IL‐23 and IL‐17 serum levels than healthy controls. CLL patients showed decreased Th1 and Th17 cytokines and increased Th2 cytokine expression. Downregulation of Th17 cells was correlated with disease progression implying a protective role of Th17 cells in CLL.^37^, ^38^ Young et al demonstrated that the progression of premalignant oral lesions to cancer in murine models was accompanied with a decline of IL‐23 and a shift in cytokine production that favoured skewing from a Th17 to an immunosuppressive Treg phenotype. Treatment with IL‐23 sustained the Th17 phenotype and slowed the progression of premalignant lesions to cancer.^39^ Various laboratory studies suggest that Th17 cells, when transferred into mice, can cause tumour regression to a greater extent than Th1 cells.^40^, ^41^ Collectively, these data suggest a potential tumour‐suppressing role of the Th17 phenotype and IL‐23, as a cytokine that polarizes CD4^+^ cells towards the Th17 phenotype in leukemia and in various solid tumours.

Furthermore, in agreement with previous studies, we detected that the lower IL‐23 levels in children with acute leukemia at diagnosis were normalized at remission, possibly attributed to cytokine changes favouring immunosuppressive T cell response and downregulating the protective one. In leukemia, an altered microenvironment in BM, PB and lymphoid tissue results from the recruitment of specific T cell subgroups and stromal cells with shifts in cytokine production.^36^, ^42^ T cell dysregulation is also commonly observed in leukemias, involving impaired cytotoxic killing of autologous tumours, altered expression of co‐stimulatory molecules and T‐subset balance, downregulation of T cell receptor signalling cascades and reduced cytokine secretion.^43^, ^44^, ^45^ CLL patients present an upregulation of the immunosuppressive Th2 and Treg cell responses and a downregulation of the protective Th1 and Th17 responses.^36^, ^42^, ^43^ This altered microenvironment of the leukemic cells in combination with the T cell dysregulation benefits the immune evasion and the expansion of the malignant clone.^42^ It is important to note that IL‐23 may exert anti‐tumorigenic effect independently of the Th17 immune response through its action on various immune cells in the tumour milieu, inducing their cytotoxic function and pro‐inflammatory cytokine production.^35^ Moreover, IL‐23 suppresses the Treg differentiation and the immune‐suppressive Treg cell response.^35^, ^46^ Furthermore, IL‐23 production by macrophages in the tumour microenvironment induces the conversion of Th17 cells into a distinct phenotype, known as non‐classical Th1 cells or Th1‐like Th17 cells that secrete IFN‐γ and promote tumour‐specific immune responses.^47^ Taking under consideration the potential anti‐tumorigenic role of IL‐23 in leukemia, the lower IL‐23 concentration in acute leukemia patients that was observed in our study could be explained as a part of the mechanisms used by the leukemic cells to modulate their microenvironment, and evade the immune responses.^42^

In our study, the initially low IL‐23 levels at diagnosis returned to normal expression pattern after the administration of the induction therapy both in ALL and AML patients. Our results support the assumption that the increased leukemic burden at diagnosis is correlated with lower IL‐23 levels and the IL‐23 expression is normalized along with the reduction of the leukemic burden. In accordance with our findings, a study of patients with pancreatic cancer showed that patients in advanced stage had lower IL‐23 and a worse Th17/Treg balance with enhanced Treg and declined Th17 cell expression while long‐term survivors had increased IL‐23 expression^48^, ^49^, ^50^ These findings support our hypothesis that the increased malignant burden is correlated with lower IL‐23 levels and that normal IL‐23 expression patterns are re‐established with the remission of the disease. Of importance, our study included a child with a blast crisis relapse of Philadelphia chromosome‐positive Chronic Myeloid Leukemia (CML), in which, IL‐23 concentration at CML diagnosis, at the chronic phase, was 40.015pg/mL while at the blast crisis, relapse was reduced to 19.973 pg/mL, reinforcing our hypothesis.

Blogowski et al demonstrated lower IL‐23 levels in pancreatic patients compared to controls which associated with the circulating number of the BM‐derived mesenchymal stem cells (BMMSCs) that support haematopoiesis and modulate immune response via cytokine secretion.^50^, ^51^ In various hematopoietic disorders, BMMSCs presented altered cytokine expression and an impaired immunoregulatory function.^52^ AML‐derived BMMSCs were more immunosuppressive/anti‐inflammatory and reduced the secretion of pro‐inflammatory cytokines, contributing to disease evolution in AML patients.^53^ In childhood ALL, the MSC niches in BM were shown to contribute to asparaginase resistance.^54^ It is possible that the leukemic cells themselves reprogramme BMMSCs to provide a niche that protects their growth and clonal evolution.^55^ The role of IL‐23 in the interaction between the leukemic cells and the MSCs has not yet been investigated. The downregulation of IL‐23 in leukemia patients may result from the interplay between the malignant cells and the BM microenvironment as part of the mechanisms used by the leukemic cells to promote their expansion.

However, the studies of IL‐23 and Th17 cells in both solid and haematological malignancies are highly inconsistent and, in many times, contradictory. Sherry et al reported that IL‐23 serum levels and Th17 cells were significantly increased in CLL patients compared to controls with significant heterogeneity in IL‐23 levels been observed in different patients.^45^ Th17 cells are elevated in CLL patients with better prognostic markers and correlate with longer survival.^56^ These observations indicate that Th17 cells and IL‐23, as a cytokine that maintains the Th17 response, favour clinical outcome.

Several studies support a pro‐tumorigenic role of IL‐23 in different cancer types. Langowski et al^57^ provided the first evidence of the tumour‐promoting effect of IL‐23, demonstrating that in murine models, the *IL‐23* deletion or blockade resulted in significant increase of CD8+ T cells infiltration with protective effect against tumour cells. IL‐23 promotes inflammatory responses, such as the upregulation of matrix metalloproteases (MMPs), angiogenesis and macrophage infiltration, while it reduces CD8+ T‐cell infiltration, and thus reduces anti‐tumour immune surveillance.^31^, ^58^ It has been proposed that, while the endogenous expression of IL‐23 appears to exert protumour activity, the exogenous overexpression of IL‐23 presents anti‐tumour effects.^22^

In our study, because of the low number of cases, IL‐23 levels did not reach any statistical correlation with the MRD result at the EIT. Therefore, a safe conclusion on the prognostic use of IL‐23 on the MRD result cannot be drawn and needs further investigation.

In summary, we detected decreased IL‐23 levels in PB and BM of children with acute leukemia, which were significantly improved at the EIT with the elimination of the leukemic burden. Taking under consideration the potential anti‐leukemic effect of IL‐23, the downregulation that was observed in our study may be involved in leukemic cell expansion. The underlying mechanisms resulting in IL‐23 downregulation are not elucidated and need further studies. Possible mechanisms involve secretion of soluble factors that may suppress specific anti‐tumour immune responses and genetic or epigenetic regulation of immune cells. A detailed study of the BM microenvironment would provide insight on the IL‐23 role in the interactions between leukemic cells and the tumour microenvironment. Given the fact that immune checkpoint receptors are a major mechanism of tumour‐induced immune suppression, their role in IL‐23 regulation is of great interest for future investigation. Immune checkpoint receptors such as PD‐1, expressed in monocytes/macrophages, have been shown to regulate IL‐23 expression in viral infections, such as hepatitis while anti‐PD‐1 treatment significantly increases IL‐23 levels.^27^, ^59^

Understanding the way that IL‐23 contributes to the control of different types of cancer, including acute leukemias of childhood, may lead to re‐evaluation of the potential therapeutic use of this molecule. IL‐12, which is the prototype cytokine of this family, is considered as a strong candidate for immunotherapy‐based interventions. In both preclinical and clinical trials, IL‐12 administration has exerted significant anti‐tumour and anti‐metastatic activity.^35^ A trial performed in low‐grade non‐Hodgkin lymphoma patients demonstrated that combined administration of IL‐12 with rituximab resulted in longer response compared to stand‐alone.^22^ However, the therapeutic effect of IL‐12 in clinical trials has been limited by systemic toxicity.^31^ The use of IL‐23 in cancer patients has not been yet investigated. However, in preclinical trials, the IL‐23 administration has showed direct anti‐tumour activity in several cancer types, including pediatric B‐ALL, exerting lower toxicity compared to IL‐12 treatment, possibly because of the lower induction of IFN‐γ.^22^ Collectively, these data suggest that IL‐23 may be a good candidate treatment to be tested in phase 1 trial in pediatric acute leukemia patients.

## CONFLICT OF INTEREST

The authors declare no conflict of interest.

## AUTHOR CONTRIBUTIONS

**Archontis Zampogiannis:** Conceptualization (equal); Formal analysis (equal); Investigation (equal); Methodology (equal); Software (equal); Validation (equal); Visualization (equal); Writing‐original draft (equal). **C. Piperi:** Writing‐original draft (equal); Writing‐review & editing (equal). **Margarita Baka:** Resources (equal); Validation (equal). **Ilianna Zoi:** Formal analysis (equal); Methodology (equal). **Athanasios G. Papavassiliou:** Writing‐review & editing (equal). **Maria Moschovi:** Conceptualization (equal); Data curation (equal); Funding acquisition (equal); Project administration (equal); Resources (equal); Supervision (equal); Validation (equal); Visualization (equal); Writing‐review & editing (equal).

## Data Availability

All data have been reported.
